# Uterine Ultrasound Doppler Hemodynamics of Magnesium Sulfate Combined with Labetalol in the Treatment of Pregnancy-Induced Hypertension Using Empirical Wavelet Transform Algorithm

**DOI:** 10.1155/2022/7951342

**Published:** 2022-05-26

**Authors:** Chunjuan Liu, Fengzhen Wang, Xueyu Yin

**Affiliations:** Department of Obstetrics, Taiyuan Maternal and Child Health Hospital, Taiyuan 030020, Shanxi, China

## Abstract

The aim of this study was to explore the hemodynamic changes of magnesium sulfate combined with labetalol in the treatment of pregnancy-induced hypertension (PIH) under Doppler uterine ultrasound based on the empirical wavelet transform (EWT) algorithm. 500 patients with PIH in the hospital were selected and randomly divided into the control group (*n* = 250) and the observation group (*n* = 250). The control group was treated with conventional magnesium sulfate; the observation group was given labetalol based on magnesium sulfate drip in the control group. The uterine artery blood flow simulation model was established based on the EWT algorithm and compared with a short-time Fourier transform (STFT). The normalized root mean square error (NRMSE) of the STFT method was 0.19, and the NRMSE extracted by the EWT method was 0.13. After treatment, the blood pressure index, 24-hour urinary protein, and incidence of adverse birth outcomes in the observation group were lower than those in the control group; the effective rate of the control group (90.4%) was lower than that of the observation group (97.6%); the hemodynamic indexes of the uterine artery in the observation group were lower than those in the control group, and the differences were statistically significant (*P* < 0.05). The estimation accuracy of the EWT method was higher than that of the traditional STFT method; the combined treatment of magnesium sulfate and labetalol in patients with PIH had a remarkable effect, which could control the blood pressure index and reduce the 24-hour urinary protein; the uterine artery Doppler ultrasound examination could change hemodynamics and improve the adverse outcomes of mothers and infants.

## 1. Introduction

Pregnancy-induced hypertension (PIH) is one of the most serious complications during pregnancy, which is characterized by high morbidity and high mortality [1]. The incidence of PIH is 9.4% in China and 7% to 12% in foreign countries [2]. Drug therapy is the main treatment for PIH, of which magnesium sulfate is the drug of choice for the treatment of PIH in clinical practice. The drug has an obvious diuretic effect and can significantly lower blood pressure and improve spasm. Pascoal et al. (2019) [3] concluded that the application of magnesium sulfate alone in the treatment of PIH would affect fetal development. Labetalol can effectively promote vasodilatation, and its antihypertensive effect is obvious, while it has little effect on the placenta. Therefore, it should be combined with labetalol while controlling the dose of magnesium sulfate to relieve the patient's clinical symptoms and improve maternal and fetal outcomes [4–6].

As one of the important parameters to evaluate human physiology and pathology, arterial hemodynamics can not only reflect the level of human metabolism but also contribute to the diagnosis of PIH disease. Therefore, the measurement of blood flow velocity distribution has a very important diagnostic value in the clinic. The ultrasonic Doppler frequency shift method is widely used in clinical hemodynamic detection because of its noninvasive and real-time advantages [7–9]. Gilles (French mathematician) combined the self-adaptability of empirical mode decomposition (EMD) with the theory of wavelet analysis and proposed the empirical wavelet transform (EWT) algorithm, which was a breakthrough in nonstationary signal processing. The core idea of the EWT algorithm is to adaptively divide the Fourier spectrum of the signal, and the instantaneous frequency and amplitude of the signal are obtained by the Hilbert transform of the decomposed single component [10–12].

In order to quantitatively analyze the ultrasonic Doppler blood flow more accurately to diagnose PIH diseases, a time-frequency analysis method based on the EWT algorithm was innovatively proposed. The time-frequency analysis method based on EWT is used to obtain better time-frequency resolution, so that the extraction of Doppler frequency shift information is more accurate and the estimation accuracy of blood flow velocity distribution is higher. The purpose is to explore the clinical effect of magnesium sulfate combined with labetalol in the treatment of PIH and to provide an experimental basis for the clinical diagnosis and treatment of PIH.

## 2. Materials and Methods

### 2.1. Study Subjects

A total of 500 patients with PIH admitted to the hospital from March 2016 to March 2020 were selected and randomly divided into the control group (*n* = 250) and the observation group (*n* = 250). The age of 250 patients in the control group was 23–38 years old, with an average of (28.4 ± 1.5) years old; gestational weeks were 28–40 weeks, with an average of (35.8 ± 1.2) weeks; there were 121 primiparas and 129 multiparas. The age of 250 patients in the observation group was 24–37 years old, with an average of (29.4 ± 1.2) years old; gestational weeks were 28–40 weeks, with an average of (35.9 ± 1.3) weeks; there were 122 primiparas and 128 multiparas. There was no significant difference in basic information such as age and gestational age between the two groups (*P* > 0.05). This study was approved by the ethics committee of the hospital. All the patients and their families signed an informed consent form.

Inclusion criteria were as follows: patients meeting the diagnostic criteria of PIH in Chinese Obstetrics and Gynecology [13] and patients with clinical symptoms such as edema, elevated blood pressure, proteinuria, and preeclampsia.

Exclusion criteria were as follows: patients with previous hypertension, diabetes, and chronic hepatitis and patients with allergy to labetalol and magnesium sulfate.

### 2.2. Treatment Methods

The patients in the control group were given routine treatment, intravenous drip of 100 mL 5% glucose injection, and 20 mL magnesium sulfate mixture, one time/day, drip time: within 30 min. The mixture of 1,000 mL 5% glucose injection and 60–80 mL magnesium sulfate was continuously pumped according to the specific conditions of the patients. Infusion time was ≤30 min to ensure that the blood magnesium ion concentration of the patients was not less than 3.0 mmol/*L*.

The observation group was given labetalol based on magnesium sulfate drip in the control group. 100 mL 5% glucose injection was mixed with 20 mL labetalol for intravenous drip, one time/day, drip time ≤30 min; drip rate maintained at 1–4 mL/min. According to the specific conditions of patients, 1,000 mL 5% glucose injection and 40–60 mL magnesium sulfate mixture were continuously pumped, and the drip time was ≤30 min.

### 2.3. Ultrasonic Detection Method

All PIH patients underwent Doppler ultrasonography. During the examination, the subjects took a supine position and kept breathing calm. A digital color Doppler ultrasound diagnostic instrument was used for examination. The abdominal exploration frequency was set to 3.5 MHz. The umbilical cord placenta end was located within 5 cm of the placental attachment point, and the bilateral uterine artery trunk was found at the distal branch of the internal iliac artery on both sides. Pulse-Doppler sampling volume was set to 2 mm^3^, and uterine artery long axis and acoustic wave angle were <60°, and at least three consecutive consistent and stable uterine artery and fetal umbilical artery blood flow maps were obtained. Resistance index (RI), pulsatile index (PI), and peak systolic velocity/end-diastolic velocity (*S*/*D*) were measured and recorded.

### 2.4. Observation Indicators

The clinical efficacy, systolic blood pressure, diastolic blood pressure, and 24-hour urinary protein change before and after treatment, and the incidence of adverse reactions in the two groups was observed and compared. Clinical efficacy criteria were as follows: proteinuria, edema, and other symptoms disappeared, and blood pressure returned to normal and cured. Proteinuria, edema, and other symptoms disappeared and blood pressure improved significantly, markedly effective. Proteinuria, edema, and blood pressure improved, effective. Elevated blood pressure, proteinuria, edema, and other symptoms did not change, even a trend of deterioration was ineffective.

### 2.5. Empirical Wavelet Transform Algorithm

The EWT normalizes the Doppler signal to [0, *π*] and divides the spectrum into *N* continuous parts to meet Λ_*n*_=[*ϖ*_*n*−1_, *ϖ*_*n*_], *n*=1,2, *N*(*ϖ*_0_=0, *ϖ*_*n*_=*π*). The EWT is constructed using Little Wood-Paley and Meyer wavelets. The constructed empirical wavelet scaling function (1) and empirical wavelet function (2) are as follows:

(1) $$\begin{array}{c} {\hat{W}}_{n}\left(\varpi \right)=\left\{\begin{array}{c} 1,\left|\varpi \right|\leq \varpi_{n}-\xi_{n},\\ \cos\left[\frac{\pi }{2}\beta \left(\frac{1}{2\xi_{n}}\left(\left|\varpi \right|-\varpi_{n}+\xi_{n}\right)\right)\right]\varpi_{n}-\xi_{n}\leq \left|\varpi \right|\leq \varpi_{n}+\xi_{n},\\ 0,\text{others,} \end{array}\right. \end{array}$$

(2) $$\begin{array}{c} \hat{w_{n}}\left(\varpi \right)=\left\{\begin{array}{c} 1,\varpi_{n}+\xi_{n}\leq \left|\varpi \right|\leq \varpi_{n+1}-\xi_{n+1},\\ \cos\left[\frac{\pi }{2}\beta \left(\frac{1}{2\xi_{n+1}}\left(\left|\varpi \right|-\varpi_{n+1}+\xi_{n+1}\right)\right)\right]\varpi_{n+1}-\xi_{n+1}\leq \left|\varpi \right|\leq \varpi_{n+1}+\xi_{n+1},\\ \begin{array}{c} \sin\left[\frac{\pi }{2}\beta \left(\frac{1}{2\xi_{n}}\left(\left|\varpi \right|-\varpi_{n}+\xi_{n}\right)\right)\right]\varpi_{n}+\xi_{n}\leq \left|\varpi \right|\leq \varpi_{n}+\xi_{n},\\ 0,\text{others.} \end{array} \end{array}\right. \end{array}$$

In the above equations, the reconstruction equation meeting *ξ*_*n*_=*κϖ*_*n*_ and *κ* < min_*n*_[*ϖ*_*n*−1_ − *ϖ*_*n*_/*ϖ*_*n*+1_+*ϖ*_*n*_] original signal can be expressed as follows:

(3) $$\begin{array}{c} r\left(t\right)=D_{r}^{\varepsilon }\left(0,t\right)\times \hat{W_{1}}\left(t\right)+\sum_{n=1}^{N}D_{r}^{\varepsilon }\left(n,t\right)\times w_{n}\left(\varpi \right). \end{array}$$

*D*_*r*_^*ε*^(0, *t*) and *D*_*r*_^*ε*^(*n*, *t*) represent the approximation function and the detail function.

*N* empirical modal functions of the original signal are obtained.

(4) $$\begin{array}{c} r_{k}\left(t\right)=D_{r}^{\varepsilon }\left(0,t\right)\times w_{n}\left(\varpi \right),k=1,2,...,N-1. \end{array}$$

### 2.6. Establishment of Uterine Artery Blood Flow Simulation Model

The commonly used hemodynamic models include the pure resistance model, elastic cavity model, and Womersley theory. The premise of using Womersley theory is that blood is a Newtonian fluid, blood is laminar and axisymmetric, the blood vessel wall is incompressible and has small deformation, and blood flow velocity is far less than pulse wave velocity.

(5) $$\begin{array}{c} -\frac{\text{d}p}{\text{d}h}={\text{Ae}}^{f}, \end{array}$$

*P* represents the pressure, *h* means the displacement in the pressure change direction, *A* represents the amplitude, and *f* means the frequency. (5) is introduced into the Navier-Stoke equation, and the solution of the Womersley theoretical equation is derived.

(6) $$\begin{array}{c} \text{W}=\frac{\pi u^{2}}{\eta }G\frac{G_{0}}{\partial^{2}}\sin\left(\omega t-\varphi +\varepsilon \right)\underset{x⟶\infty }{\lim}, \end{array}$$

$\partial =u\sqrt{\omega /\eta }$ is named as the Womersley number or frequency parameter. *u* represents hemodynamic viscosity, and *G* means arterial pressure gradient constant. *G*_0_, *ε*, *∂* indicate parameters related to frequency. If the blood flow velocity is a constant value, it indicates that the radial velocity distribution of blood flow in the lumen is as follows:

(7) $$\begin{array}{c} V\left(y\right)=2v_{0}\left(1-r^{2}\right), \end{array}$$

*v*_0_ represents the blood flow velocity in the center of the lumen, and *r* means the ratio of radial position to lumen radius. According to (7), it can be inferred that the blood flow velocity is distributed according to the radial position of the blood vessel; that is, the blood flow at the same radial position has the same velocity, and the flow velocity gradually decreases from the lumen center to the tube wall.

The accuracy of the time-frequency analysis method in estimating the blood flow velocity distribution of the uterine artery is studied, so the computer simulation of establishing the physical model of blood flow tissue is helpful to understand the Doppler signal more comprehensively, so as to facilitate the subsequent analysis and research of hemodynamics.

Because the position of the point on the cross-sectional area of each section of the blood vessel is different, its hemodynamics is also different. If the length is too large, the subsequent analysis may not be detailed. Therefore, the selected volume is small to fully highlight the complexity of hemodynamics in blood vessels. According to the space size of the scattering point, ultrasonic wavelength, and 10 scattering point particles in each cube wavelength, the least number of scattering point particles that should be scattered in the circular tube can be inferred.

(8) $$\begin{array}{c} N=10\times \frac{xyz}{\lambda^{3}}, \end{array}$$

*λ* represents the ultrasonic wavelength, which meets *λ*=*c*/*f*_0_. *c* represents the wave velocity of ultrasonic propagation in the tissue, and *f*_0_ represents the center frequency of the transducer.

Radial distribution of particle velocity of scattering point in arterial blood flow tissue simulation model is as follows:

(9) $$\begin{array}{c} v\left(l\right)=v_{0}\left(p1-{\left(\frac{l}{L}\right)}^{2}\right), \end{array}$$

*v*_0_ means the velocity of particles at the scattering point at the center of the lumen, which meets the *v*_0_=1*m*/*s*. *l* represents the distance from the scattering point particle to the center of the lumen. *L* is the lumen radius.

The simulation of blood flow Doppler signal is expressed as the linear addition of sinusoidal signal.

(10) $$\begin{array}{c} U=\sum_{j}\vartheta_{j}\cos\left[\varphi_{d}\left(t\right)+\phi_{j}\right], \end{array}$$

*ϑ*_*j*_ represents the transmission and scattering characteristics of ultrasound, *φ*_*d*_(*t*) denotes the Doppler frequency shift, and *ϕ*_*j*_ indicates the random phase subject to the uniform distribution.

### 2.7. Verification of Simulation Experiment

Blood flow signal has the characteristics of nonstationary random, and short-time Fourier transform (STFT) is usually used for time-frequency analysis. This method can accurately extract the characteristic signal, which is a widely used nonlinear nonstationary signal analysis method. STFT has good time-frequency resolution and can extract feature signals well, but it cannot balance frequency resolution and time resolution. In order to quantitatively analyze the accuracy of STFT and EWT methods for estimating blood flow velocity, the normalized root mean square error (NRMSE) between the blood flow velocity profile and the theoretical value is calculated by the two methods.

(11) $$\begin{array}{c} NRMSE=\frac{\sqrt{\sum_{k-1}^{m}{\left(v\left(k\right)-\overset{-}{v}\left(k\right)\right)}^{2}}}{\sqrt{\sum_{k-1}^{m}{\left(\overset{-}{v}\left(k\right)\right)}^{2}}}, \end{array}$$

$\overset{-}{v}\left(k\right)$ denotes the theoretical velocity value corresponding to each point, *v*(*k*) represents the estimated value of blood flow velocity obtained from the analysis of Doppler blood flow tissue signal, and *m* means the sample size, *m*=500.

### 2.8. Statistical Methods

The data were analyzed by SPSS19.0 statistical software. The measurement data were expressed as mean ± standard deviation ($\bar{x}$ ± *s*), and the count data were expressed as percentage (%). The difference was statistically significant with *P* < 0.05.

## 3. Results

### 3.1. Results of Simulation Experiment

**Figure 1:**
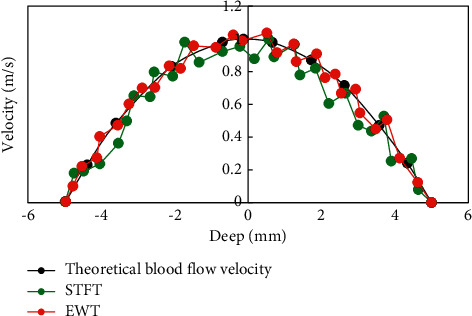
Blood flow velocity.

**Figure 2:**
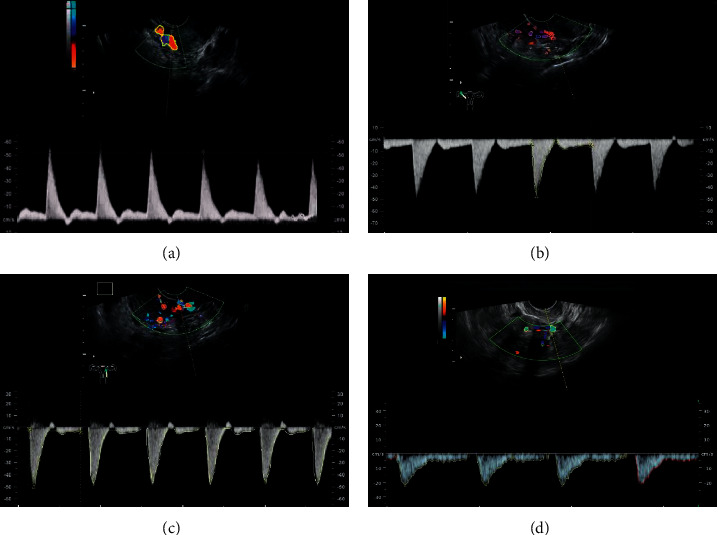
Ultrasound image during pregnancy. (a) Normal uterine artery ultrasound of normal pregnant women; (b) abnormal uterine artery ultrasound of normal pregnant women; (c) abnormal uterine artery under ultrasound based on STFT algorithm; (d) abnormal uterine artery under ultrasound based on EWT algorithm.

**Figure 3:**
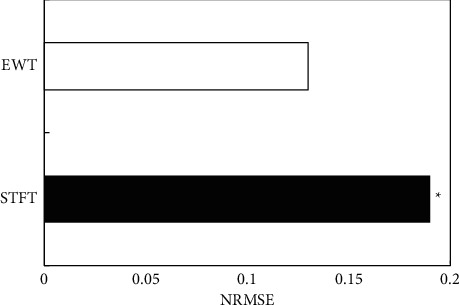
Comparison of NRMSE between two algorithms. ^*∗*^Compared with the EWT algorithm, *P* < 0.05.

In order to test the accuracy of the STFT and EWT methods in estimating blood flow velocity, two methods to estimate uterine artery blood flow velocity profile were measured (Figure 1). The black curve represented the theoretical velocity, the green curve was the blood flow velocity estimated by the traditional STFT method, and the red curve was the blood flow velocity estimated through the Doppler blood flow signal after dividing the frequency band by the EWT algorithm. The blood flow velocity profile estimated by the EWT method was in good agreement with the theoretical velocity profile compared with the STFT method. Ultrasound based on the EWT algorithm to determine abnormal uterine artery blood flow velocity was more accurate (Figure 2). The NRMSE of the STFT method was 0.19, and the NRMSE extracted by the EWT method was 0.13. The EWT method had higher estimation accuracy than the traditional STFT method (Figure 3).

### 3.2. Comparison of Blood Pressure Indexes before and after Treatment between the Two Groups

**Figure 4:**
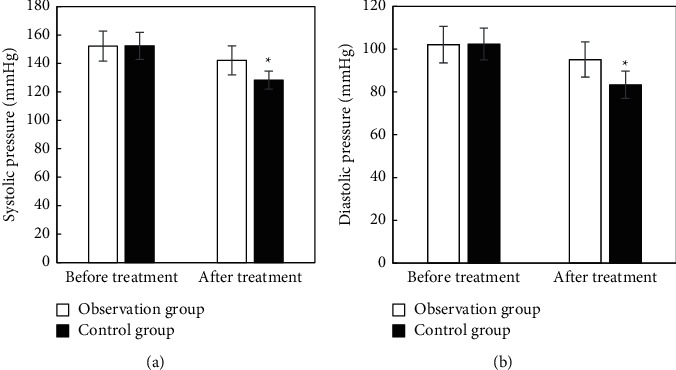
Comparison of blood pressure indexes before and after treatment between two groups. (a) Systolic blood pressure; (b) diastolic blood pressure. ^*∗*^Compared with the observation group, *P* < 0.05.

There was no significant difference in systolic and diastolic blood pressure between the two groups before treatment (*P* > 0.05). After treatment, the systolic and diastolic blood pressure in the observation group was significantly lower than that in the control group, and the difference between the two groups was significant, with statistical significance (*P* < 0.05) (Figure 4).

### 3.3. Doppler Ultrasound

**Figure 5:**
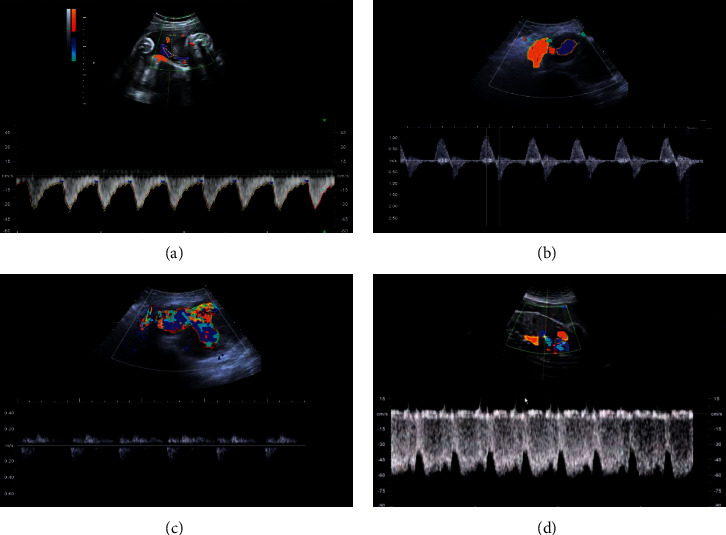
Blood flow spectrum. (a) Umbilical artery; (b) aortic stenosis; (c) fetal PV; (d) fetal venous catheter.

The tissue Doppler imaging mode was initiated. The umbilical artery (UA) was located from the placenta umbilical cord connection port to the fetal abdominal umbilical cord entrance. The aortic isthmus (AoI) was located at the aortic arch of the three vessels and trachea section of the fetal heart (the angle between the vessel and the Doppler ultrasound beam was 0–30°), and the pulmonary vein (PV) was located at the four-chamber section of the fetal chest (the angle between the vessel and the Doppler ultrasound beam was <20°). The fetal ductus venosus (DV) was located on the transverse section of the fetal abdomen (the angle between the blood vessel and Doppler ultrasound beam was <30°) (Figure 5).

### 3.4. Comparison of Clinical Efficacy between the Two Groups of Patients with PIH

The comparison of the efficacy between the two groups showed that the number of cured, significantly effective, effective, and ineffective patients in the control group was 106, 89, 31, and 24, respectively, with an effective rate of 90.4%; the number of cured, significantly effective, effective, and ineffective patients in the observation group was 156, 74, 14, and 6, with an effective rate of 97.6%, and the difference had statistical significance (*P* < 0.05) (Table 1).

### 3.5. Changes in 24-Hour Urinary Protein in the Two Groups

**Figure 6:**
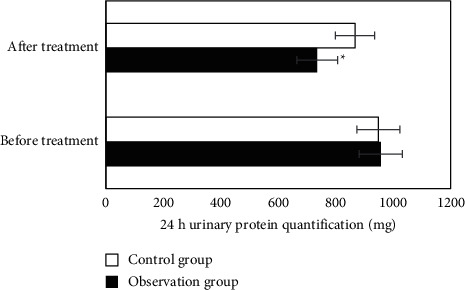
Changes in 24-hour urinary protein in the two groups. ^*∗*^Compared with the control group, *P* < 0.05.

Before treatment, there was no significant difference in 24-hour urinary protein between the two groups (*P* > 0.05); after treatment, the 24-hour urine protein content of the observation group (735.26 ± 68.25) mg was significantly lower than that of the control group (867.13 ± 75.34) mg, and there was a significant difference between the two groups (*P* < 0.05) (Figure 6).

### 3.6. Comparison of Delivery Outcomes between the Two Groups

The incidence rate of adverse delivery outcomes in the observation group was significantly lower than that in the control group, with statistical significance (*P* < 0.05) (Table 2).

### 3.7. Differences in Hemodynamic Indexes of Uterine Artery between the Two Groups

The hemodynamic indexes RI, PI, and S/*D* of the uterine artery in the observation group were significantly lower than those in the control group, with statistical significance (*P* < 0.05) (Table 3).

## 4. Discussion

PIH often occurs in pregnant women after 20 weeks of pregnancy, and the patients are generally primiparas. The main clinical manifestations are systemic small artery spasm, which leads to stenosis of the vascular lumen and abnormal operation of the blood circulation system, leading to the continuous rise of blood pressure indicators until systemic coma, headache and dizziness, and systemic edema occur. In severe cases, it may even cause adverse pregnancy outcomes such as stillbirth [14–16].

Ultrasonic Doppler blood flow velocity measurement is an important technology in ultrasonic medicine. It completes the diagnosis of human diseases by analyzing the velocity information carried by the extracted blood flow echo signal. At present, the traditional Doppler method is most used for clinical blood flow velocity measurement [17]. Doppler blood flow echo signal contains human disease diagnosis information. The traditional time-frequency analysis method based on STFT and the time-frequency analysis method based on the EWT algorithm were compared and analyzed. STFT cannot provide a good compromise effect of the time-frequency resolution, and when the window function width is determined, the time-frequency resolution is determined. To change the time-frequency resolution, it is necessary to change the size of the window function, which limits its application in engineering practice. The EWT method extracts the characteristic signal after adaptive decomposition of the signal, which has good time-frequency resolution and is not limited, and is more suitable for the analysis of nonlinear and nonstationary signals [18, 19]. The NRMSE of the STFT method is 0.19, and the NRMSE extracted by the EWT method is 0.13. The estimation accuracy of the EWT method is higher than that of the traditional STFT method.

Magnesium sulfate is clinically recognized as the preferred drug for the treatment of PIH. It can act on the location of the peripheral vascular neuromuscular junction, inhibit the release of acetylcholine, reduce the content of acetylcholine, effectively inhibit the release of calcium ions, and reduce the content of calcium ions. After treatment, the 24-hour urine protein content of the observation group (735.26 ± 68.25) mg was significantly lower than that of the control group (867.13 ± 75.34) mg (*P* < 0.05); the RI, PI, and *S*/*D* of the uterine artery in the observation group were significantly lower than those in the control group (*P* < 0.05), indicating that the combined use of magnesium sulfate and labetalol can reduce the hemodynamic parameters of the uterine artery, effectively treat PIH, and improve the delivery outcome of patients and the prognosis of mothers and infants. The study of Shepherd et al. [20] showed that the antihypertensive effect of magnesium sulfate alone was remarkable, but the speed was slow. The control effect on patients with severe or acute diseases was poor or the disease could not be controlled in time, and the delivery outcome and prognosis could not be effectively improved. Therefore, it could be combined with other drugs. Therefore, in order to improve the antihypertensive effect and improve the delivery outcome, clinical treatment should actively explore effective means. Li et al. [21] believed that the combination of magnesium sulfate and labetalol had an obvious therapeutic effect on PIH, which could effectively improve the delivery outcome and prognosis. This is similar to the result of this study.

## 5. Conclusion

The uterine blood flow tissue model based on the EWT algorithm was first constructed, and magnesium sulfate combined with labetalol was used to treat PIH patients under the guidance of Doppler ultrasound. The combined treatment of magnesium sulfate and labetalol in patients with PIH has a remarkable effect, which can effectively control the blood pressure index and reduce the 24-hour urinary protein. The effect of uterine artery Doppler ultrasound examination is remarkable, which can accurately detect fetal heart function and change hemodynamics and improve maternal and infant adverse outcomes. There are certain deficiencies. Due to the limitation of time, there is a lack of long-term follow-up for patients, so long-term follow-up for patients is needed in the later stage to further verify the long-term efficacy. It provides some ideas and experimental support for the diagnosis and treatment of PIH patients.

## Figures and Tables

**Table 1:** Comparison of clinical efficacy between the two groups of patients with PIH (*n*, %).

Group	Patients with cured	Patients with significantly effective	Patients with effective	Patients with ineffective	Total effective cases
Control group (*n* = 250)	106 (42.4%)	89 (35.6%)	31 (12.4%)	24 (9.6%)	226 (90.4%)
Observation group (*n* = 250)	156 (62.4%)	74 (29.6%)	14 (5.6%)	6 (2.4%)	244 (97.6%)
*χ* ^2^	18.425	8.652	7.562	6.158	7.568
*P*	<0.05	<0.05	<0.05	<0.05	<0.05

**Table 2:** Comparison of delivery outcomes between the two groups (*n*, %).

Group	Cervical laceration	Fetal distress	Neonatal asphyxia	Postpartum hemorrhage	Total
Control group (*n* = 250)	8 (3.2%)	9 (3.6%)	11 (4.4%)	15 (6%)	29 (11.6%)
Observation group (*n* = 250)	3 (1.2%)	2 (0.8%)	4 (1.6%)	7 (2.8%)	16 (6.4%)
*χ* ^2^	1.475	2.358	3.142	2.671	2.547
*P*	<0.05	<0.05	<0.05	<0.05	<0.05

**Table 3:** Differences in hemodynamic indexes of the uterine artery between the two groups ($\bar{x}$ ± *s*).

Group	RI	PI	*S*/*D*
Control group (*n* = 250)	0.64 ± 0.12	1.26 ± 0.35	3.67 ± 0.46
Observation group (*n* = 250)	0.55 ± 0.14	0.78 ± 0.18	2.78 ± 0.67
*T*	5.672	10.463	10.081
*P*	<0.05	<0.05	<0.05

## Data Availability

The data used to support the findings of this study are available from the corresponding author upon request.
